# The ratio of Th17/Treg cells as a risk indicator in early acute respiratory distress syndrome

**DOI:** 10.1186/s13054-015-0811-2

**Published:** 2015-03-11

**Authors:** Zhi-xin Yu, Mu-sen Ji, Jun Yan, Yan Cai, Jing Liu, Hong-feng Yang, Yong Li, Zhao-chen Jin, Jin-xu Zheng

**Affiliations:** Department of Critical Care Medicine, The Affiliated People’s Hospital, Jiangsu University, Zhenjiang, 212002 China; Department of Respiratory Medicine, Affiliated Hospital of Jiangsu University, Zhenjiang, 212001 China

## Abstract

**Introduction:**

Recent studies have revealed that lung inflammation mediated by CD4+ T cells may contribute to the pathogenesis of acute respiratory distress syndrome (ARDS). The imbalance between CD4 + CD25 + Foxp3 + regulatory T (Treg) cells and T helper (Th)17 cells has been found in a number of different inflammation and autoimmune diseases, while the role of the Th17/Treg balance in ARDS remains largely unknown. The aim of this study was to investigate the Th17/Treg pattern and its impact on disease severity and outcomes in patients with ARDS.

**Methods:**

This prospective, observational study enrolled 79 patients who fulfilled the Berlin definition of ARDS and 26 age- and sex-matched healthy controls. Circulation Th17 and Treg cell frequencies were analyzed by flow cytometry, and the expressions of Th17- and Treg-related cytokines in serum were measured by enzyme-linked immunosorbent assay (ELISA). Acute Physiologic and Chronic Health Evaluation (APACHE) II score, Sequential Organ Failure Assessment (SOFA) score, and the Lung Injury Score were also calculated at enrollment.

**Results:**

Within 24 hours after the onset of ARDS, the changes of peripheral circulating Th17 and Treg cell frequencies gradually increased from mild to severe ARDS. Th17/Treg ratio was positively correlated with APACHE II score, SOFA score, and Lung Injury Score, while negatively correlated with PaO_2_/FiO_2_. The areas under the receiver operating characteristic (AUC) curves of Th17/Treg ratio for predicting 28-day mortality in ARDS patients was higher than that of APACHE II score, SOFA score, Lung injury score, as well as PaO_2_/FiO_2_. Using a Th17/Treg ratio cutoff value of >0.79 to determine 28-day mortality, the sensitivity was 87.5% with 68.1% specificity. Multivariate logistic regression showed Th17/Treg ratio >0.79 (odds ratio = 8.68, *P* = 0.002) was the independent predictor for 28-day mortality in patients with ARDS. Finally, cumulative survival rates at 28-day follow-up also differed significantly between patients with Th17/Treg ratio >0.79 and ≤0.79 (*P* <0.001).

**Conclusions:**

The Th17/Treg imbalance favoring a Th17 shift represents a potential therapeutic target to alleviate lung injury and a novel risk indicator in patients with early ARDS.

## Introduction

Acute respiratory distress syndrome (ARDS) is a life-threatening clinical syndrome of rapid-onset pulmonary failure with high morbidity and mortality in critically ill patients [1,2]. Despite decades of intense research, the pathogenesis leading to this devastating disease remains poorly defined. However, clinical and animal studies have shown that activation of multiple inflammatory cells and release of inflammatory mediators play an important role in the development and outcome of ARDS [1]. Among those cells, immune cells including neutrophils [3], macrophages [4] and dendritic cells [5] involved in this process have been studied extensively. Most recently, the involvement of lymphocytes, especially CD4+ T cells, in the pathogenesis of ARDS has become an active topic of research [6-12].

CD4 + CD25 + Foxp3 + regulatory T (Treg) cells have an anti-inflammatory role mainly by contact-dependent suppression or releasing cytokines, IL-10 and transforming growth factor (TGF)-β1 on other immune cells, including CD4+ and CD8+ T cells, B cells, natural killer (NK) cells and dendritic cells [13]. Numerous publications have revealed that reduced generation or deficient function of Treg cells, which is associated with disease severity and activity, has been found in patients with various inflammation and autoimmune diseases [13]. In mice and patients with acute lung injury (ALI), the alveolar recruitment of Treg cells, specifically mediated by leukotriene B4 (LTB4)-leukotriene B4 Receptor (BLT1) pathway [10], contribute to the resolution of lung inflammation [7], particularly in the resolution of ALI fibroproliferation [9]. However, data from a recent observational clinical study showed that an increased Treg cell ratio in bronchoalveolar lavage fluid (BALF) obtained from ARDS patients on admission is an independent risk factor for 30-day mortality [8]. Therefore, the precise role of Treg cells on ARDS and its resolution remains to be explored.

In contrast to Treg cells, a more recently discovered effector subset of CD4+ T cells, Th17 cells play a potent proinflammatory role in the immune system through producing the signature cytokines IL-17 and to a lesser extent other inflammatory mediators including IL-6, TNF-α, granulocyte-macrophage colonystimulating factor [13]. In addition to their effector function in defense against extracellular pathogens, T helper (Th)17 cells can promote many autoimmune inflammatory conditions including several lung diseases (asthma [14], tuberculosis [15], lung cancer [16], and chronic obstructive pulmonary disease [17]), unless they are efficiently controlled by regulatory cells [13]. Remarkably, Th17 development relies on the key cytokine TGF-β1, which is also linked to Treg cell development and function, indicating that Th17 and Treg not only exert opposite functions in the immune response but also share reciprocal development pathways. Moreover, under certain inflammatory environments, differentiated Treg cells have a tendency to be reprogrammed and converted into Th17 cells [13,18]. Thus, it is reasonable to hypothesize that the fine balance between Th17 and Treg is crucial for maintenance of immune homeostasis.

The imbalance of Th17/Treg has also been found in a number of different inflammation and autoimmune diseases [13]. Although the role of Th17 cells in the development and progress of ARDS is currently unclear, IL-17, the cytokine preferentially produced by Th17 cells, has been widely investigated in lung diseases including ARDS [19]. Furthermore, Th17 cells have been implicated in other lung diseases in animal experimentation and clinical studies [20]. In addition to Th17 cells found in the BALF fluid of patients with respiratory diseases, two studies have shown that losartan and alanyl-glutamine may protect mice from lipopolysaccharide-induced lung injury by suppressing Th17 immune responses and modulating the Th17/Treg balance in favor of Treg cells, respectively [5,12]. Therefore, we hypothesize that the imbalance of Th17/Treg might exist and play a role in the pathogenesis of inflammation in ARDS. To test this hypothesis, we investigated the distribution of Th17 cells in relation to Treg cells in ARDS patients, and evaluated how the imbalance of Th17/Treg and their related cytokines correlate with the disease severity.

## Materials and methods

### Subjects and protocol

This prospective, observational study was conducted in the 30-bed, level-3 multi-disciplinary, adult ICU of the Affiliated People’s Hospital, Jiangsu University. Between June 2012 and January 2014, consecutive patients admitted to our ICU were considered eligible if they met the Berlin definition of ARDS [2]. Patients were classified according to the arterial partial pressure of oxygen/inspired oxygen fraction (PaO_2_/FiO_2_) ratio as mild (200 mmHg < PaO_2_/FiO_2_ ≤ 300 mmHg, n = 24), moderate (100 mmHg < PaO_2_/FiO_2_ ≤ 200 mmHg, n = 28), and severe (PaO_2_/FiO_2_ ≤ 100 mmHg, n = 27). The exclusion criteria were as follows: age <18 years, known history of cancer, end-stage liver or renal disease, and chronic immune-mediated disorders. At the time of sample collection, none of the patients had received any corticosteroids, or other non-steroid anti-inflammatory drugs. In addition, we also excluded the patients who died within 24 h of receiving a diagnosis of ARDS.

Finally, after excluding 12 patients who failed to meet the inclusion criteria and 7 patients who were lost to follow up, 79 patients were enrolled and were also categorized as survivors (n = 47) and non-survivors (n = 32) according to clinical outcome within 28 days of follow up. Twenty-six age- and sex-matched healthy volunteers without a history of any chronic disease served as controls. This study was approved by the Ethics Committee of the Affiliated People’s Hospital, Jiangsu University. Informed consent from all of the subjects or their relatives was obtained before enrollment.

### Data collection and outcome

After enrollment, demographics and baseline characteristics such as age, sex, body mass index (BMI), risk factors for ARDS, severity of illness at ICU admission (acute physiologic and chronic health evaluation (APACHE) II score) [21], sequential organ failure assessment (SOFA) score [22], and the lung injury score [23] were also calculated after ICU admission. All patients were followed up for 28 days. The main outcomes were mortality at discharge from ICU and from hospital at 28 days.

### Blood sampling and measurements

Within 24 h after diagnosis of ARDS, peripheral whole blood samples were collected in tubes containing heparin or ethylene diamine tetraacetate acid (EDTA), and serum was obtained after centrifugation and stored at −80°C until used. White blood cell count, C-reactive protein (CPR) and procalcitonin (PCT) were immediately measured in the clinical chemistry laboratory of the Affiliated People’s Hospital. The serum levels of TGF-β1, IL-6, IL-10, and IL-17 (all from R&D Systems, Minneapolis, MN) were determined by enzyme-linked immunosorbent assay (ELISA), following the manufacturer’s instructions.

### Flow cytometric analysis

Peripheral blood mononuclear cells (PBMCs) were freshly isolated by Ficoll density gradient centrifugation (Pharmacia, Uppsala, Sweden). The isolated PBMCs were washed twice with PBS) and resuspended at 1 × 10^6^ cells/mL in complete culture medium (RPMI 1640 supplemented with 1% penicillin/streptomycin, 2 mM _L_-glutamine, and 10% heat-inactivated fetal bovine serum; Gibco BRL, Gaithersburg, MD, USA).

For Th17 cells analysis, IL-17-producing CD3+ lymphocytes were detected as described previously [24]. PBMCs were stimulated for 4 h with 2 μ/mL leukocyte activation cocktail (BD Pharmingen™, USA) at 37°C and 5% CO_2_. Upon harvest, cells were then washed twice using PBS, and surface-stained with APC-conjugated anti-CD8 (BD Pharmingen™, USA) and PerCP-conjugated anti-CD3 (BD Pharmingen™, USA) for 20 minutes at room temperature in the dark. Following surface staining, cells were incubated with PE-conjugated anti-IL-17A (BD Pharmingen™, USA) after fixation and permeabilization using the IntraPrep Permeabilization Reagent (Beckman Coulter Inc., France).

For the analysis of Treg cells, a human regulatory T-cell staining kit (BD Pharmingen™, USA) was used to measure CD4 + CD25 + Foxp3+ cells as Treg cells according to the manufacturer’s protocol. Briefly, PBMCs was incubated with a cocktail of fluorescein isothiocyanate-conjugated anti-CD4 and APC-conjugated anti-CD25 for 30 minutes at 4°C. After fixation and permeabilization, the cells were blocked by normal rat serum and stained using PE-conjugated anti-Foxp3 for 45 minutes at 4°C.

The flow cytometry analyses were performed on a FACSCalibur flow cytometer (BD biosciences, San Jose, CA, USA) equipped with CellQuest software (BD biosciences). Isotype controls were conducted to ensure antibody specificity.

### Statistical analysis

All analyses were done using SPSS, version 20.0 (IBM Corp., Armonk, NY, USA) and MedCalc version 11.0 (MedCalc Software, Inc, Mariakerke, Belgium). Continuous variables were reported as mean ± SD or as median (IQR) after testing their normal distribution using the Kolmogorov-Smirnov test.

For two-group comparisons, the independent samples *t*-test was used for normally distributed data, and the Mann-Whitney test was used for non-normally distributed data. For the comparison of multi-group, one-way analysis of variance and the Kruskal-Wallis test was used to analyze normally and non-normally distributed data, respectively, and *P*-values were adjusted with the Bonferroni correction for multiple comparisons. Categorical data are summarized using number (percentage), and were compared using the chi-square or Fisher’s exact test. Spearman’s rank correlation was applied to determine the correlation between variables.

The area under the receiver operating characteristic (ROC) curve was calculated to evaluate the diagnostic and prognostic value of the tested parameters. The cutoff points were calculated by acquiring the best Youden index (sensitivity + specificity − 1). Kaplan-Meier plots and the log-rank test were used to compare survival in several groups. To determine the independent predictors of 28-day mortality, binary logistic regression analysis was performed as stepwise regression (entering a variable if *P* was <0.05, removing a variable if *P* was >0.10) for variables with a *P*-value of <0.05. Results were expressed as the odds ratio (OR), *P*-value and 95% CI.

All tests were two-tailed, and a value of *P* <0.05 was considered statistically significant. In addition, *P* <0.008 (after Bonferroni correction) was used for multiple comparisons.

## Results

### Baseline characteristics and patient outcome

Between June 2012 and January 2014, a total of 79 patients who fulfilled the Berlin definition of ARDS and 26 healthy volunteers were included in this study. Tables 1 and 2 show the characteristics at enrollment and outcomes of the study population. No significant differences were found in age, gender and body mass index (BMI) among the four groups (mild, moderate, severe ARDS, and control groups). Pneumonia, sepsis and trauma were the most common etiology of ARDS. As shown in Tables 1 and 2, severity of critical illness on the day of enrollment, measured by the APACHE II score (*P* <0.001), SOFA score (*P* <0.001), lung injury score (*P* <0.001) and PaO_2_/FiO_2_ (*P* <0.001), worsened from mild to severe ARDS. The 28-day mortality was 40.5% (32/79) in patients with ARDS. Compared with survivors, non-survivors had significantly higher APACHE II and SOFA scores. In addition, survivors had higher PaO_2_/FiO_2_ and lower lung injury score than non-survivors.

**Table 1:** **Baseline characteristics of the population enrolled in the study**

**Variables**	**Control**	**Acute respiratory distress syndrome**				^**¶**^ ***P*** **-value**
		**Total**	**Mild**	**Moderate**	**Severe**	
Number	26	79	24	28	27	
Age, years	54.00 ± 13.77	55.03 ± 14.42	55.17 ± 15.45	53.36 ± 13.34	57.63 ± 15.57	0.714
Gender, male/female, n	14/12	44/35	13/11	15/13	16/11	0.971
BMI, kg/m^2^	22.44 ± 3.53	24.03 ± 4.13	24.72 ± 4.98	24.67 ± 3.84	24.26 ± 3.93	0.150
Cause of ARDS						
Pneumonia		25	6	9	10	
Non-pulmonary sepsis		19	4	8	7	
Trauma		23	9	6	8	
Aspiration		5	2	2	1	
Multiple transfusions		4	2	2		
Others		3	1	1	1	
APACHE II score		22 (20 to 26)	20 (18 to 22)	23 (21 to 26)*	25 (22 to 27)*	<0.001
SOFA score		11.0 (10.0 to 13.0)	9.5 (7.0 to 11.8)	11.0 (10.0 to 13.0)*	12.0 (11.0 to 13.0)*	<0.001
Lung injury score		2.3 (2.1 to 2.8)	2.0 (1.9 to 2.1)	2.3 (2.1 to 2.5)*	2.9 (2.7 to 3.2)*^#^	<0.001
PaO_2_/FiO_2_ (mmHg)		138 (76 to 222)	258 (223 to 273)	139 (120 to 158)*	67 (59 to 79)*^#^	<0.001
CRP, mg/L	4.97 (3.83 to 6.12)	135.84 (116.83 to 156.79)	116.69 (104.17 to 133.33)	130.97 (123.40 to 154.45)	156.85 (143.59 to 185.36)*	<0.001
PCT, ng/mL	0.04 (0.02 to 0.06)	1.56 (1.24 to 1.83)	1.24 (1.05 to 136)	1.47 (1.27 to 1.66)	2.04 (1.62 to 2.61)*^#^	<0.001
Leukocytes, 10^9^/L	6.8 (5.6 to 8.2)	12.9 (11.2 to 14.8)	11.8 (10.5 to 12.8)	12.4 (10.7 to 13.7)	15.3 (13.6 to 17.6)*^#^	<0.001
Monocytes, 10^9^/L	0.58 (0.46 to 0.69)	1.05 (0.92 to 1.23)	0.95 (0.84 to 1.13)	1.04 (0.88 to 1.23)	1.12 (1.04 to 1.1.26)*	<0.001
Lymphocytes, 10^9^/L	3.09 (2.80 to 3.52)	5.35 (4.25 to 6.47)	4.24 (3.93 to 4.96)	5.24 (4.19 to 6.06)*	6.72 (5.69 to 7.45)*^#^	<0.001
Th17 cells, % of CD4^+^	0.71 (0.59 to 0.94)	4.27 (3.67 to 6.02)	3.54 (3.17 to 3.94)	4.16 (3.96 to 4.54)*	6.42 (5.96 to 6.91)*^#^	<0.001
Treg cells, % of CD4^+^	3.11 (2.70 to 3.44)	5.37 (4.98 to 6.07)	4.82 (4.50 to 5.18)	5.46 (5.25 to 5.81)*	6.18 (5.82 to 6.57)*^#^	<0.001
Th17/Treg ratio	0.25 ± 0.09	0.86 ± 0.20	0.74 ± 0.12	0.77 ± 0.10	1.01 ± 0.18*^#^	<0.001
IL-6, pg/mL	11.70 ± 1.31	758.33 ± 70.03	721.53 ± 54.43	763.62 ± 76.96*	785.56 ± 62.36*	<0.001
IL-17, pg/mL	20.27 ± 4.12	199.59 ± 68.12	139.42 ± 25.51	204.75 ± 37.64*	247.72 ± 78.08*^#^	<0.001
IL-10, pg/mL	123.04 ± 20.53	131.19 ± 19.94	137.8 ± 19.17	132.49 ± 20.14	124.17 ± 18.87	0.029
TGF-β1, pg/mL	193.48 ± 46.48	209.92 ± 51.00	219.30 ± 61.84	200.30 ± 41.77	211.55 ± 49.25	0.264
28-day mortality, n, %		32 (40.5%)	7 (29.17%)	12 (42.86%)	13 (48.15%)	

Quantitative data with a normal distribution are presented as mean ± SD. Quantitative data with a non-normal distribution are presented as median (IQR). Qualitative data are presented as number (%). ^¶^ *P*-value for the four groups (mild, moderate, severe ARDS, and control groups); *P <0.01 versus mild ARDS; ^#^ *P* <0.01 versus moderate ARDS. BMI, body mass index; APACHE, acute physiologic and chronic health evaluation; SOFA, sequential organ failure assessment; CRP, C-reactive protein; PCT, procalcitonin.

**Table 2:** **Comparison of clinical characteristics of patients with acute respiratory distress syndrome (ARDS) according to survival**

**Variables**	**Non-survivors (n = 32)**	**Survivors (n = 47)**	***P*** **-value**
Age, years	53.38 ± 13.80	56.72 ± 15.27	0.323
Gender, male/female, n	17/15	26/21	0.848
BMI, kg/m^2^	24.34 ± 4.12	24.69 ± 4.29	0.716
APACH II score	26 (22 to 27)	21 (19 to 23)	<0.001
SOFA score	12 (11 to 13)	10 (9 to 12)	<0.001
Lung injury score	2.6 (2.2 to 3.1)	2.1 (2.0 to 2.7)	0.002
PaO_2_/FiO_2_, mmHg	111 (67 to 183)	157 (84 to 231)	0.037
CRP, mg/L	138.59 (123.40 to 165.47)	135.64 (116.53 to 156.76)	0.413
PCT, ng/mL	1.64 (1.33 to 2.05)	1.38 (1.07 to 1.67)	0.031
Leukocytes, 10^9^/L	13.2 (11.5 to 15.8)	12.7 (10.8 to 14.5)	0.507
Monocytes, 10^9^/L	1.07 (0.99 to 1.28)	1.04 (0.88 to 1.18)	0.124
Lymphocytes, 10^9^/L	5.76 (4.80 to 6.61)	5.10 (4.16 to 6.21)	0.044
Th17 cells, % of CD4^+^	4.84 (4.25 to 6.76)	4.01 (3.56 to 5.34)	<0.001
Treg cells, % of CD4^+^	5.33 (4.98 to 5.94)	5.46 (4.98 to 6.12)	0.583
Th17/Treg ratio	0.98 ± 0.20	0.78 ± 0.15	<0.001
IL-6, pg/mL	790.68 ± 66.95	736.30 ± 63.80	<0.001
IL-17, pg/mL	229.09 ± 77.18	179.50 ± 53.25	0.003
IL-10, pg/mL	128.48 ± 21.87	133.04 ± 18.53	0.321
TGF-β, pg/mL	216.81 ± 55.44	205.23 ± 47.80	0.325

Quantitative data with a normal distribution are presented as mean ± SD. Quantitative data with a non-normal distribution are presented as median (IQR). BMI, body mass index; APACHE, acute physiologic and chronic health evaluation; SOFA, sequential organ failure assessment; CRP, C-reactive protein; PCT, procalcitonin.

### Alteration of inflammatory biomarkers, circulating Th17 and Treg cells and their associated cytokines in ARDS patients

The changes in inflammatory biomarkers, Th17 and Treg cell frequencies and their associated cytokine levels in each group are shown in Tables 1 and 2. Compared to the healthy control group, the levels of CRP and PCT, and the counts of leukocytes, monocytes, and lymphocytes were markedly elevated in ARDS patients (*P* <0.001) (Table 1). PCT levels and leukocyte counts were higher in patients with severe ARDS than in patients with mild or moderate ARDS, and patients with severe ARDS also had higher CPR levels and monocytes count as compared to patients with mild ARDS (*P* <0.01) (Table 1). Interestingly, the lymphocyte count increased as the severity of ARDS increased from mild to severe (*P* <0.01) (Table 1). Furthermore, non-survivors had higher PCT levels and lymphocyte counts than did survivors, but the CRP levels and leukocyte and monocyte counts were similar in the two groups (Table 2).

As shown in Table 1, the frequencies of Th17 and Treg cells were evidently increased in the peripheral blood of ARDS patients than those in the control group (*P* <0.001). Moreover, the changes in Th17 and Treg cell frequencies progressively increased with ARDS severity from mild to moderate and severe ARDS (*P* <0.01). However, although the frequencies of Th17 cells in non-survivors were higher as compared to those in survivors, there was no significant difference between two groups in frequencies of Treg cells (Table 2). Given the changes in these two types of immune cells, we used the Th17/Treg ratio to describe their relationship to further investigate their functions and differentiation. The Th17/Treg ratio was significantly higher in patients with ARDS when compared with the control group (*P* <0.001, Table 1). Furthermore, patients with severe ARDS had a higher Th17/Treg ratio than patients with mild and moderate ARDS (*P* <0.01) (Table 1). In non-survivors, a highly significant Th17/Treg ratio was also found in favor of a proinflammatory Th17-response (*P* <0.001) (Table 2).

Th17-related cytokines (IL-6, IL-17) and Treg-related cytokines (IL-10, TGF-β1) were detected by means of ELISA (Tables 1 and 2). IL-6 and IL-17 were significantly higher compared with those in the control group (*P* <0.001) (Table 1), and both these cytokines were associated with the severity or progression of ARDS, as non-survivors had significantly higher IL-6 and IL-17 levels (*P* <0.001, *P* = 0.003, respectively) (Table 2). While IL-10 levels was increased in ARDS patients when compared to those in the control group (*P* = 0.029) (Table 1), there was no significant difference in the TGF-β1 levels among all groups (*P* = 0.264) (Table 1). In addition, levels of both IL-10 and TGF-β1 were similar between survivors and non-survivors (*P* = 0.321, *P* = 0.325, respectively) (Table 2).

### Correlation of Th17/Treg ratio with disease severity and outcome

**Figure 1:**
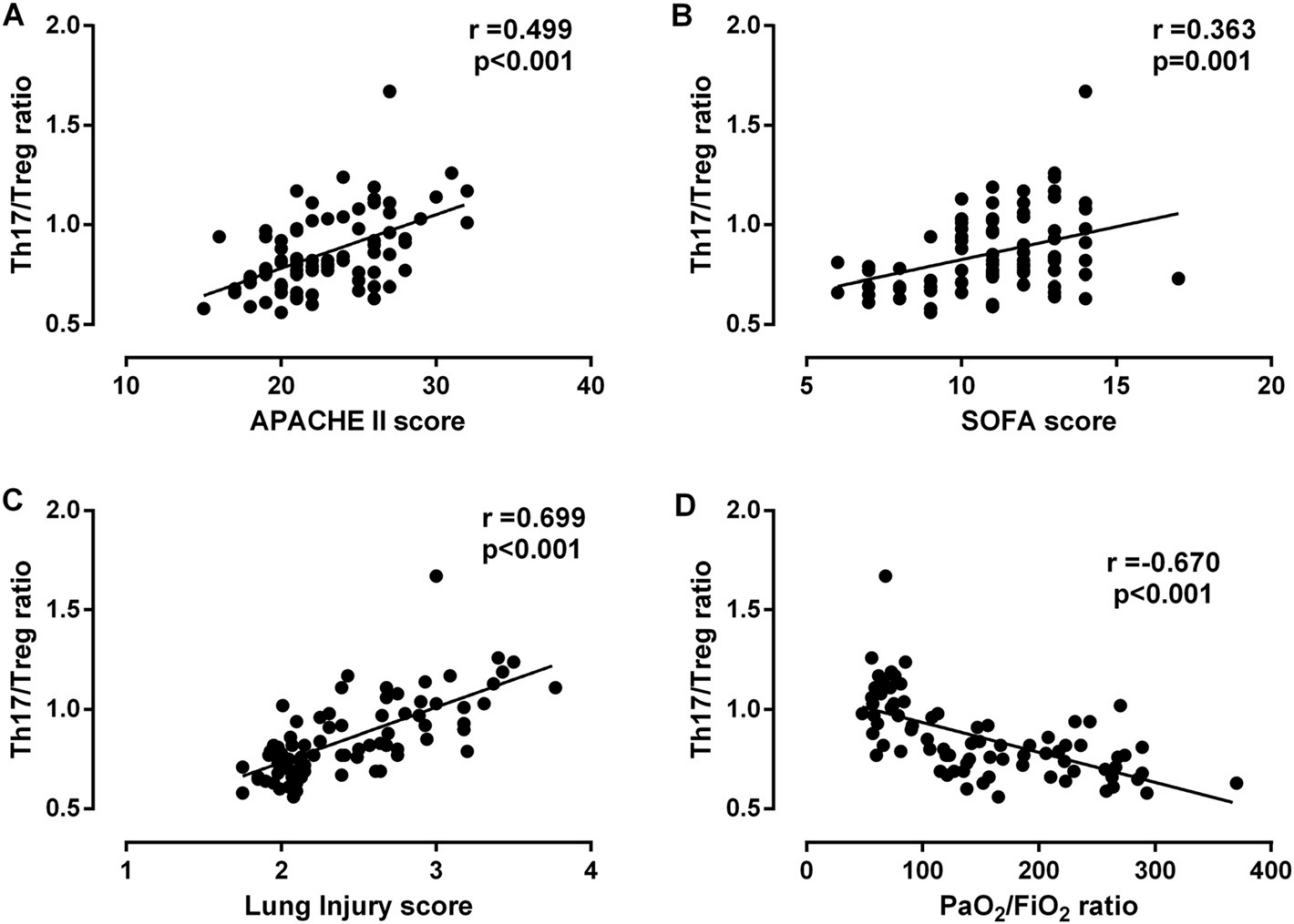
**Relationship between ratio of Th17/Treg cells and acute physiology and chronic health evaluation (APACHE) II, sequential organ failure assessment (SOFA) or lung injury score, or arterial partial pressure of oxygen/inspired oxygen fraction (PaO**_**2**_ **/FiO**_**2**_ **) in patients with acute respiratory distress syndrome (ARDS).** Spearman rank correlation was tested between variables. The ratio of Th17/Treg cells was positively correlated with APACHE II score **(A)**, SOFA score **(B)**, and lung injury score **(C)**, while it was negatively correlated with PaO_2_/FiO_2_ **(D)** in ARDS patients.

Spearman correlation analysis of Th17/Treg ratio with APACHE II score, SOFA score, lung injury score, and PaO_2_/FiO_2_ in ARDS patients are displayed in Figure 1. For all patients with ARDS, the significantly positive and moderate correlations were found between Th17/Treg ratio and APACHE II score (*r* = 0.499, *P* <0.001), SOFA score (*r* = 0.363, *P* = 0.001), Lung injury score (*r* = 0.699, *P* <0.001), respectively. Furthermore, we noticed a negative and moderate correlation between the Th17/Treg ratio and PaO_2_/FiO_2_ (*r* = −0.670, *P* <0.001).

**Figure 2:**
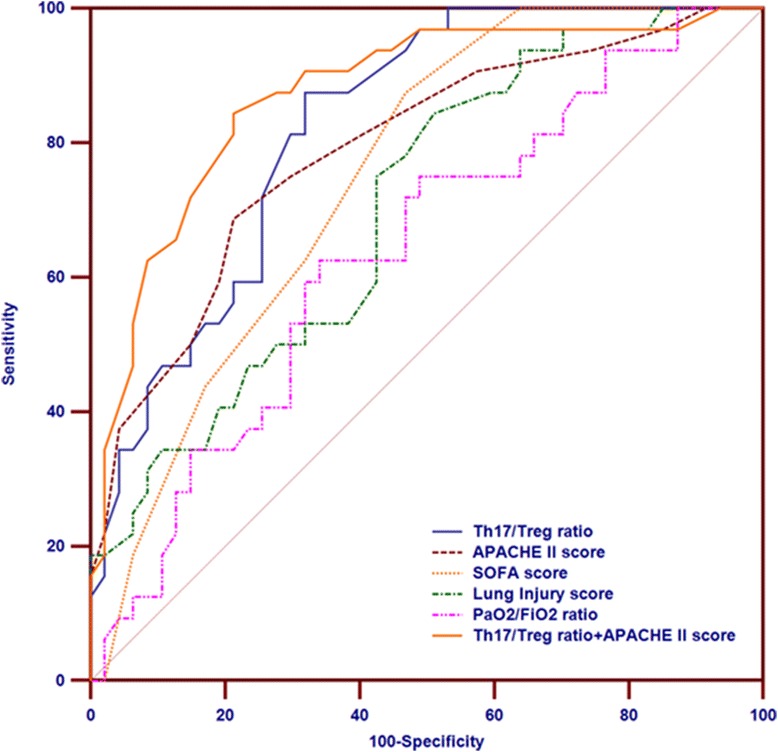
**Receiver operating characteristic (ROC) curves for the Th17/Treg ratio, acute physiology and chronic health evaluation (APACHE) II, sequential organ failure assessment (SOFA) and lung injury scores, arterial partial pressure of oxygen/inspired oxygen fraction (PaO**_**2**_ **/FiO**_**2**_ **), and Th17/Treg ratio in combination with APACHE II score for predicting 28-day mortality in patients with acute respiratory distress syndrome (ARDS).** The area under the curve (AUC) demonstrates that the Th17/Treg ratio measures 0.824 (95% CI 0.722 to 0.901), the APACHE II score measures 0.791 (95% CI 0.684 to 0.874), the SOFA score measures 0.749 (95% CI 0.639 to 0.840), the lung injury score measures 0.704 (95% CI 0.590 to 0.801), PaO_2_/FiO_2_ measures 0.639 (95% CI 0.523 to 0.744) and the Th17/Treg ratio in combination with the APACHE II score measures 0.872 (95% CI 0.790 to 0.954).

The ROC curves for Th17/Treg ratio, APACHE II score, SOFA score, lung injury score, PaO_2_/FiO_2_ and Th17/Treg ratio in combination with APACHE II score for predicting 28-day mortality in ARDS patients are shown in Figure 2. The area under the ROC curve (AUC) of Th17/Treg ratio for predicting 28-day mortality in ARDS patients was 0.824 (95% CI 0.722 to 0.901), higher than that for the APACHE II score (0.791, 95% CI 0.684 to 0.874), but this was not statistically significant (*P* = 0.558). Compared to the Th17/Treg ratio, the AUC for the lung injury score (0.704, 95% CI 0.590 to 0.801) was significantly lower (*P* = 0.011); the AUC for the SOFA score was also lower (0.749, 95% CI 0.639 to 0.840), but this difference did not reach statistical significance (*P* = 0.257). Moreover, the AUC for PaO_2_/FiO_2_ for 28-day mortality was 0.639 (95% CI 0.523 to 0.744) compared to the AUC for the Th17/Treg ratio, APACHE II score, SOFA score, and lung injury score (*P* = 0.0001, 0.01, 0.132, and 0.063, respectively). The AUC for the Th17/Treg ratio in combination with the APACHE II score was 0.872 (95% CI 0.790 to 0.954), which was significantly higher than that for the APACHE II score alone for predicting 28-day mortality (*P* = 0.034), and there was no difference for the combination of the Th17/Treg ratio and APACHE II score compared with the Th17/Treg ratio alone (*P* = 0.171).

Using a Th17/Treg ratio cutoff value of >0.79 for predicting 28-day mortality in patients with ARDS, the sensitivity and specificity was 87.5% and 68.1%, respectively, and the positive and negative likelihood ratios were 2.74 and 0.18.

### Predictors for 28-day mortality in patients with ARDS

**Figure 3:**
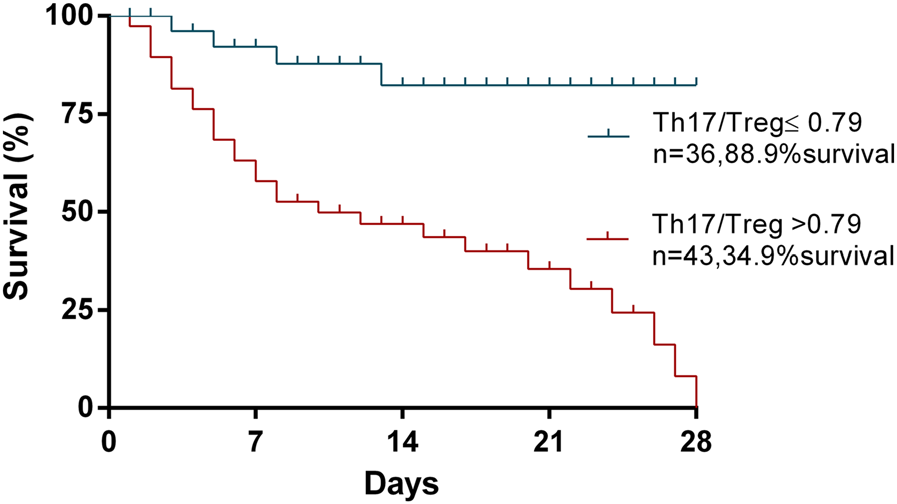
**Kaplan-Meier survival curve for patients with ARDS using the cutoff value of Th17/Treg ratio based on receiver operating characteristic (ROC) analysis.** Log-rank test (*P* <0.001).

Table 3 shows that the Th17/Treg ratio (OR = 8.68, *P* = 0.002), APACHE II score (OR = 1.32, *P* = 0.007), and SOFA score (OR = 1.49, *P* = 0.030) were the independent predictors of 28-day mortality in patients with ARDS. Moreover, ARDS patients with a Th17/Treg ratio >0.79 had higher 28-day mortality (*P* <0.001, Figure 3).

**Table 3:** **Logistic regression analysis of mortality prediction for patients with acute respiratory distress syndrome (ARDS)**

**Variables**	**Univariate analysis**		**Multivariate analysis**	
	**Odds ratio (95% CI)**	***P*** **-value**	**Odds ratio (95% CI)**	***P*** **-value**
APACHE II score, per point	1.42 (1.19, 1.68)	<0.001	1.32 (1.08, 1.62)	0.007
SOFA score, per point	1.62 (1.22, 2.15)	0.001	1.49 (1.04, 2.15)	0.030
Lung injury score, >2.1	5.18 (1.70, 15.74)	0.004		
PaO_2_/FiO_2_, per log_10_ (mmHg)	0.14 (0.02, 1.00)	0.051		
PCT, per log_10_ (ng/mL)	2.38 (0.98, 5.78)	0.056		
Lymphocytes, >5.24 × 10^9^/L	2.97 (1.15, 7.64)	0.024		
Th17 cells, >4.12% of CD4^+^	8.70 (2.84, 26.69)	<0.001		
IL-6, >794.63 pg/mL	5.53 (1.97, 15.48)	0.001		
IL-17, >188.56 pg/mL	3.88 (1.49, 10.09)	0.005		
Th17/Treg ratio, >0.79	14.93 (4.44, 50.28)	<0.001	8.68 (2.25, 33.54)	0.002

APACHE, acute physiologic and chronic health evaluation; SOFA, sequential organ failure assessment; PCT, procalcitonin.

## Discussion

It is generally believed that inflammatory cells and their associated mediators are mandatory in the pathological process of ARDS. Over the past few decades, despite extensive studies on these cells and mediators in both humans and mice, the pathogenesis of ARDS remains poorly understood [1,3,4,25]. Recently, it has been suggested that T lymphocytes, especially CD4+ T cells, contribute to the progress of autoimmune and inflammation diseases, including ARDS [1,5-12]. In this study, we demonstrated that the Th17/Treg balance in the peripheral blood of patients with ARDS changed toward a Th17 response compared to the control group. Moreover, the circulating Th17/Treg ratio of non-survivors was significantly higher than that of survivors. The ratio of Th17/Treg cells was also closely related to the severity of illness and 28-day mortality in ARDS patients. Thus, the skewing of the Th17/Treg ratio may contribute to the pathogenesis of ARDS.

Prior studies have shown that Treg cells, as important regulators of immune response mainly though cell-to-cell contact and secretion of the inhibitory cytokines IL-10 and TGF-β1, infiltrate the lung and may be involved in pathogenesis of ARDS. D’Alessio *et al*. showed for the first time that Treg cells accumulate in the BALF of mice and patients with ALI and contribute to the resolution of ALI by inducing TGF-β1 and neutrophil apoptosis [7]. They further showed that Treg cells reduce ALI fibroproliferation through control of fibrocyte recruitment to the lung after lipopolysaccharide-induced injury [9]. Moreover, Wang *et al*. demonstrated that the well-known proinflammatory LTB4-BLT1 pathway may contribute to the resolution of ALI by mediating the alveolar recruitment of Treg cells [10]. In addition to these studies that were mainly performed in mice with ALI, in a recent observational clinical study the researchers reported that an increased ratio of CD4 + CD25 + Foxp3 + Treg cells to all CD4+ cells in BALF obtained from ARDS patients on admission is an independent risk factor for 30-day mortality, although the ratio of Treg to all CD4+ cells in the blood was not associated with 30-day mortality and did not differ between patients with ARDS and controls [8]. However, in the current study, the Treg cell percentage in peripheral blood was significantly higher in patients with ARDS than in the control group. Furthermore, the changes in Treg cell frequencies progressively increased with ARDS severity from mild to moderate and severe ARDS. The frequency of Treg cells in survivors tended to be higher, although this trend did not reach statistical significance. The difference may be largely due to the different study groups. Adamzik *et al*. only studied ARDS cases caused by community-acquired pneumonia, whereas we recruited a mixed population including patients with pulmonary and extrapulmonary ARDS. Indeed, ARDS is a heterogeneous syndrome that can occur as a result of multiple insults and diseases. It is noteworthy that the differences between pulmonary and extrapulmonary ARDS may exist not only in morphology and respiratory physiology but also in response to therapeutic interventions, despite the fact that a common end state may be present [26].

As a more recently discovered effector subset of CD4+ T cells, Th17 cells play a key role in defense against extracellular pathogens, and promote many autoimmune inflammatory conditions [13]. Although the role of Th17 cells has been investigated in several lung diseases, such as asthma [14], tuberculosis [15], lung cancer [16], and chronic obstructive pulmonary disease [17], there are few data about the peripheral frequency of Th17 cells in ARDS patients. In our study, we found that ARDS patients exhibited a significant increase in the frequency of Th17 cells and their signature cytokine, IL-17, compared to the control group. Moreover, a similar significant difference was observed between survivors and non-survivors. These results suggest a potential role for circulating Th17 cells to reflect the severity of lung injury.

Th17 cells and Treg cells not only share reciprocal development pathways but also are mutually opposite in function. Naïve CD4+ T cells differentiate into distinct functional subsets account to the local cytokine environment. Moreover, under certain inflammatory milieu, differentiated Treg cells can be converted into Th17 cells [13,18]. TGF-β alone induces the differentiation into Treg cells, but in an IL-1- and/or IL-6-rich inflammatory milieu, the Th17 generation is enhanced while the Treg cells are inhibited [18]. The elevated levels of IL-6 found in ARDS patients and non-survivors compared to the control group and survivors, respectively, are in line with the mechanism by which Th17 cells are promoted while Treg cells are suppressed. The Th17/Treg balance toward Th17 cells might enhance the local accumulation of inflammatory mediators, and ultimately create a pathogenic proinflammatory loop to amplify proinflammatory responses in patients with ARDS.

The balance between Th17 and Treg cells has been suggested as a new paradigm for a number of different inflammation and autoimmune diseases [13]. Given the non-synchronous changes in Th17 and Treg cells, we used Th17/Treg to define the relationship between inflammation status and regulatory condition of immune system. Our study demonstrated that in peripheral blood the Th17/Treg ratio increased in ARDS patients compared with the healthy controls. The Th17/Treg ratio was also higher in patients with severe ARDS as compared to those with mild or moderate ARDS. In addition, a highly significant Th17/Treg ratio was found in favor of a proinflammatory Th17-response in non-survivors. Correction of the Th17/Treg imbalance may help maintain the immune system in a steady state, and exhibit a therapeutic benefit for this disease. Indeed, two studies have shown that losartan and alanyl-glutamine may protect mice from lipopolysaccharide-induced lung injury by suppressing Th17 immune responses and modulating the Th17/Treg balance in favor of Treg cells, respectively [5,12].

Although clinical scores, such as the APACHE II [21], SOFA [22] and lung injury score [23] have been widely used in clinical practice to predict outcome in critically ill patients, the early risk-stratification of these patients and their prognosis, as well as accurate monitoring of the effects of clinical treatment, remain a crucial challenge in critical care. Recently, the new Berlin definition has emphasized the notion that ARDS mortality markedly increased with greater severity of hypoxemia as assessed with PaO_2_/FiO_2_ [2]. In the present study, negative and moderate correlations were observed between the Th17/Treg ratio and PaO_2_/FiO_2_, indicating that the higher the Th17/Treg ratio, the more adverse the outcome in ARDS patients. Interestingly, although APACHE II score, SOFA score, and lung injury score all had significantly positive correlations with the Th17/Treg ratio, the Spearman’s correlation coefficient was the highest between the Th17/Treg ratio and lung injury score, which is more suited to discriminate pulmonary-specific outcomes [23,27]. Meanwhile, we further found that the AUC for Th17/Treg ratio for predicting 28-day mortality in ARDS patients was higher than that for the APACHE II score, SOFA score, lung injury score or PaO_2_/FiO_2_. However, the AUC for the lung injury score and PaO_2_/FiO_2_ were lower, likely because they are measures of initial lung injury severity and never intended as a prognostic tool in ARDS. Although the Th17/Treg ratio in combination with the APACHE II score slightly increased the AUC for predicting 28-day mortality, there was no difference compared with the Th17/Treg ratio alone. Moreover, the Th17/Treg ratio, APACHE II score, and SOFA score were found to be independent predictors of 28-day mortality in ARDS patients, and those patients with a ratio of Th17/Treg >0.79 had a higher mortality rate. Taken together, our findings strongly suggest that the Th17/Treg ratio is a potential indicator for the degree of lung injury and a good index for evaluation of prognosis in ARDS patients.

### Limitations

There were some limitations to this study. First, this is a single-center study and the sample size was not large, thus restricting generalizability. However, our data would be representative enough to observe the alteration of circulating Th17 and Treg cells and the correlation between the Th17/Treg ratio with disease severity and outcome. Moreover, enrolled patients are a mixed population of ARDS, including pulmonary and extrapulmonary ARDS. Further studies with larger numbers of patients are required to confirm our findings. Second, we just investigated the changes in Th17 and Treg cells from peripheral blood. Ideally, samples should be taken either from the alveolar spaces or lung tissue where the inflammatory events are occurring. However, samples obtained through bronchoalveolar lavage and/or biopsies are limited by complexity, especially among critically ill patients in ICU. More importantly, the alteration of circulating Th17 and Treg cells is present in ARDS, and is associated with increased disease severity and risk of mortality, indicating that our method is safe and feasible. Third, the change in functional immunocompetent cells was only measured at one time point, because we intended to focus on understanding the effect of the early, inflammatory phase of ARDS on clinical outcome. However, the resolution phase is also important for us to more comprehensively understand the pathophysiological process occurring in ARDS. Therefore, further studies are warranted to longitudinally measure the dynamic changes in the immunological status of patients during the whole time course of ARDS. Finally, this study was not designed to assess the association between the Th17/Treg ratio and the development of ARDS, and this will be the focus of our next study.

## Conclusions

In summary, Th17 cells and Treg cells increased in the peripheral blood of patients with early ARDS, and higher Th17 to Treg cell ratio may be associated with poorer prognosis. Moreover, strategies designed to restore the Th17/Treg balance may be a novel and effective therapeutic approach in ARDS.

## Key messages

- In patients with early ARDS, the peripheral circulating Th17 and Treg cells and their associated cytokines increased
- The Th17/Treg ratio towards the Th17 cell subset contributes to the pathogenesis of ARDS. The Th17/Treg imbalance may represent a potential therapeutic target and risk indicator in early ARDS.

## Abbreviations

ALI: acute lung injury
APACHE II score: acute physiologic and chronic health evaluation II score
ARDS: acute respiratory distress syndrome
AUC: area under the curve
BALF: bronchoalveolar lavage fluid
BMI: body mass index
CRP: C-reactive protein
EDTA: ethylene diamine tetraacetate acid
ELISA: enzyme-linked immunosorbent assay
NK: natural killer
OR: odds ratio
PaO_2_/FiO_2_: arterial partial pressure of oxygen/inspired oxygen fraction
PBMC: peripheral blood mononuclear cell
PCT: procalcitonin
PBS: phosphate-buffered saline
ROC: receiver operating characteristics
SOFA score: sequential organ failure assessment score
TGF: transforming growth factor
Th cell: T helper cell
TNF: tumor necrosis factor
Treg cell: regulatory T cell
